# Molecular Mechanisms of Synaptic Plasticity 2.0: Dynamic Changes in Neurons Functions, Physiological and Pathological Process

**DOI:** 10.3390/ijms241612685

**Published:** 2023-08-11

**Authors:** Giuseppina Martella

**Affiliations:** 1 Laboratory of Neurophysiology and Plasticity, IRCCS Fondazione Santa Lucia, 00143 Rome, Italy; martella@med.uniroma2.it or g.martella@hsantalucia.it; 2 Department of Humanistic Sciences, Faculty of Motor Sciences, Pegaso University, 80143 Naples, Italy

Due to the success of the first Special Issue on synaptic plasticity, I endeavored to promote a new Special Issue with an emphasis on dynamic changes in neuronal functions and physiological and pathological processes.

I have endorsed this Special Issue with the aim of collecting scientific research capable of intensifying readers’ interest in phenomena related to synaptic plasticity.

Synaptic plasticity is a crucial molecular mechanism whose actions are carried out from the developmental period to old age. Described briefly, our brain is subject to structural and functional modifications in response to external stimuli. Synaptic plasticity phenomena include macroscopic changes and cortical remapping in response to injury, microscopic changes, and spine pruning [1,2]. The emergency resulting from the increase in neuropsychiatric and neurological disorders over the last few years has stressed the urgency of understanding the aberrant processes connected to synaptic plasticity failure [3,4,5].

The authors invited to contribute to this Special Issue have provided important contributions consisting of the translational and clinical studies that I am pleased to promote in these few lines. This Special Issue contains two original clinical articles, two literature reviews, and five original translation articles. I hope that I have provided you with an extensive thematic collection and that you enjoy your reading.

Patnaik and co-workers examined 29 patients with certified bipolar disorder, 32 patients affected by cerebellar neurodegenerative pathologies, and 37 healthy subjects using the 3T-MRI technique in order to determine the similarities and differences in cerebellar grey matter loss. They found a pattern of grey matter cerebellar alterations in both the bipolar and cerebellar groups that involved the anterior and posterior cerebellar regions, demonstrating the involvement of the cerebellum in the synaptic plasticity of patients with bipolar disorder [6].

In multiple sclerosis, inflammation can modify synaptic transmission and plasticity. Professor Centonze’s group explored the influence of proinflammatory cytokines on associative Hebbian synaptic plasticity. In their cohort, they found that IL-1β levels were associated with synaptic hyperexcitability and inversely related to LTP-like synaptic plasticity.

These findings support the evidence that anti-IL-1β drugs represent a new potential therapeutic target. Considering the identification of IL-1β as a marker of inflammatory synaptopathy, antagonistic drugs may also represent a specific target during the different phases of the progression of multiple sclerosis [7].

In their systematic review, Eltokhi and coworkers underline the correlation between neuropsychiatric disorders and deficits in the glutamatergic system, and they also consider the psychiatric features of neurodevelopmental disorder as well as autism. Alterations in synaptic plasticity, accompanied by structural modifications of excitatory synapses, were observed in schizophrenia and autism spectrum disorders using EM-imaging methods. In addition, it was revealed that the expression of glutamatergic receptors is differentially affected in various brain regions, thus revealing an undeniable link between altered synaptic plasticity and psychiatric illness [8].

D’Angelo and colleagues investigated the A2A receptor’s role in different areas inside of a DYT1 mouse model of dystonia. They showed that A2A receptors are significantly improved in the striatal and globus pallidus nuclei and reduced in the entopeduncular nucleus. These opposite modifications may suggest that the pathophysiology of dystonia is correlated with an imbalance of the direct or indirect pathway [9].

The motor thalamus (MTh) is involved in the basal ganglia cortical loop and acts on the codifying of motor information. Di Giovanni and his co-workers showed for the first time that acute dopamine depletion caused by tetrodotoxin (TTX) results in an increase in GABA concentration in the MTh along with unchanged glutamate levels. Chronic dopamine denervation via 6-hydroxydopamine (6-OHDA) in anesthetized rats affects the coupling of MTh cortical activity in relation to the TTX-induced acute depletion state. The authors’ findings demonstrate that the MTh, among other areas in the basal ganglia, is influenced by DA chronic deprivation and makes alterations in the basal ganglia network in relation to counterbalancing the profound alteration arising after the onset of the acute DA depletion state [10].

La Recchiuta aimed to emphasize the role of the amygdala and medial prefrontal cortex in functional plasticity and synaptic wiring in conditions of fear extinction. Her results demonstrated that the optogenetic activation of pyramidal neurons in mice conditioned by induced fear extinction deficits causes an increase in cellular excitability, excitatory neurotransmission, and spinogenesis and is also associated with modifications of the transcriptome of amygdala pyramidal neurons [11].

The brain-derived neurotrophic factor (BDNF) drives brain development and maturation. Altered BDNF levels have been observed in many neurological diseases to such an extent that new therapeutic strategies are being developed to increase the level of BDNF.

Fingolimod-phosphate (FTY720-P) can modulate BDNF levels. However, the mechanisms by which the FTY720-P operates are still unclear. Patnaik and colleagues have shown that FTY720-P can regulate dendritic architecture, increase dendritic spine density, and modify the morphology of mature primary hippocampal neuron cultures. This study confirms that BDNF-dependent therapies may represent a new goal for many neurological diseases [12].

The Ghiglieri group, on the other hand, carried out scientific work demonstrating that food restriction can improve the lifespan of different species.

According to their analyses, food is a natural reward, and where this reformulation is restricted, some neuroadaptive responses are necessary to maintain physiological homeostasis.

They examined the AMPA receptor subunit composition in the dorsal striatal neurons of mice that had been acutely food deprived and showed that even moderate food deprivation in experimental animal models reflects a series of neuroadaptations and the remodeling of striatal synaptic plasticity [13].

Stroke is a great enemy of modern medicine. Usually, deficiencies of the neurovascular unit caused by reperfusion lesions, or inflammatory processes, constitute the main field of study. In their literature review, De Luca and collaborators provided a road map that could help to improve both therapy and rehabilitation through the knowledge of translational studies. They also suggest that further research should involve the cellular capacity to avoid neuroinflammatory phenomena and the capacity of cells during reperfusion to actively reshape the matrix [14].
